# The Emerging Role of Long Non-coding RNAs and Circular RNAs in Coronary Artery Disease

**DOI:** 10.3389/fcvm.2021.632393

**Published:** 2021-02-23

**Authors:** Soudeh Ghafouri-Fard, Mahdi Gholipour, Mohammad Taheri

**Affiliations:** ^1^Urogenital Stem Cell Research Center, Shahid Beheshti University of Medical Sciences, Tehran, Iran; ^2^Department of Medical Genetics, Shahid Beheshti University of Medical Sciences, Tehran, Iran; ^3^Urology and Nephrology Research Center, Shahid Beheshti University of Medical Sciences, Tehran, Iran

**Keywords:** long non-coding RNA, circRNA, coronary artery disorder, expression, biomarkers

## Abstract

Coronary artery disease (CAD) is a common disorder caused by atherosclerotic processes in the coronary arteries. This condition results from abnormal interactions between numerous cell types in the artery walls. The main participating factors in this process are accumulation of lipid deposits, endothelial cell dysfunction, macrophage induction, and changes in smooth muscle cells. Several lines of evidence underscore participation of long non-coding RNAs (lncRNAs) and circular RNAs (circRNAs) in the pathogenesis of CAD. Several lncRNAs such as H19, ANRIL, MIAT, lnc-DC, IFNG-AS1, and LEF1-AS1 have been shown to be up-regulated in the biological materials obtained from CAD patients. On the other hand, Gas5, Chast, HULC, DICER1-AS1, and MEG3 have been down-regulated in CAD patients. Meanwhile, a number of circRNAs have been demonstrated to influence function of endothelial cells or vascular smooth muscle cells, thus contributing to the pathogenesis of CAD. In the current review, we summarize the function of lncRNAs and circRNAs in the development and progression of CAD.

## Introduction

Coronary artery disease (CAD) is a common disorder caused by atherosclerotic processes in the coronary arteries. This condition can be asymptomatic or can result in fatal situations. In fact, CAD is the main cause of the mortality associated with coronary heart disorders (1). Atherosclerosis is regarded as a progressive inflammatory condition during which oxidative, hemodynamic, and biochemical factors destruct the function of endothelial cells (2). Subsequent alterations in the permeability of endothelial cells, accumulation of macrophages, production of inflammatory substances, and activation of smooth muscle cells are additional steps in the development of atherosclerosis (3, 4). Two classes of regulatory non-coding RNAs, namely, long non-coding RNAs (lncRNAs) and circular RNAs (circRNAs), have been shown to affect the process of atherosclerosis and CAD development (5, 6). Although both having regulatory effects on the expression of genes, they vary in terms of biogenesis and mechanism of action. LncRNAs have sizes of more than 200 nucleotides (7) and can function as signal, sequester, scaffold, guide, or enhancer RNAs to influence genomic organization or gene expression (8). They share several features with mRNAs such as the presence of RNA polymerase II binding sites, 3′ poly A tails and 5′ caps (9). On the other hand, circRNAs are single-stranded covalently enclosed molecules made via back-splicing of linear precursor transcripts (10). Both classes of transcript can influence function of endothelial cells or smooth muscle cells in the process of atherosclerosis. In the current review, we summarize the function of lncRNAs and circRNAs in the development and progression of CAD.

## LncRNAS and CAD

### Role of LncRNAs in CAD

LncRNAs can affect CAD pathogenesis through regulation of immune responses, modulation of function of endothelial cells and vascular smooth muscles, and changing lipid metabolism. In some cases, a certain lncRNA can affect more than one route.

### LncRNAs Regulate Immune Responses

H19 is a transcript encoded by a conserved imprinted gene cluster containing the insulin-like growth factor 2 gene (11). H19 has been shown to function as a molecular sponge for let-7. Expression of this lncRNA has been shown to be reduced in the muscle of patients with type-2 diabetes, as well as animal model of this disorder. The consequent up-regulation of let-7 decreases expression levels let-7 targets (12). This lncRNA has been overexpressed in patients with CAD despite its normal expression in other forms of cardiovascular disorders and therefore has been suggested as a marker for prediction of CAD. Notably, its expression levels have been associated with duration of CAD and serum concentrations of transforming growth factor β1 (TGF-β1). *In vitro* studies verified the effects of H19 overexpression in enhancement of TGF-β1 secretion (13). The lncRNA CoroMarker has been shown to be functionally clustered with genes, which are related with signal transduction, transmembrane transport, synaptic communication, and innate immune responses, while having negative correlation with inflammation-related genes. Small interfering RNA–mediated silencing of CoroMarker has reduced the production of proinflammatory cytokines (14). IFNG-AS1 is another overexpressed lncRNA in CAD patients whose expression has been considerably associated with Gensini score, as well as levels of inflammatory markers high sensitivity C-reactive protein (hs-CRP), tumor necrosis factor α (TNF-α), and interleukin 6 (IL-6). On the other hand, IFNG-AS1 levels have been inversely associated with the levels of anti-inflammatory cytokine IL-10 level (15). Cho et al. (16) have demonstrated DQ485454 as the main ANRIL transcript in the endothelial cells. Expression of this transcript has been significantly higher in endothelial cells compared with THP-1 monocytes. Notably, they reported down-regulation of DQ485454 in CAD coronary arteries as compared with samples obtained from non-CAD arteries. Forced up-regulation of this transcript has attenuated cellular processes participating in CAD initiation as it decreased monocyte adhesion to endothelial cells, transendothelial monocyte migration, and endothelial cell migration. Moreover, expression of several CAD-related genes were altered after DQ485454 silencing (16). A microarray-based study has demonstrated down-regulation of NEXN-AS1 in human atherosclerotic plaques. This lncRNA interacts with the chromatin modifier BAZ1A and the 5′ part of the NEXN gene. Overexpression of NEXN-AS1 suppressed TLR4 oligomerization and nuclear factor κB (NF-κB) function, decreased endothelial production of adhesion proteins and inflammatory cytokines, and repressed adhesion of monocyte to endothelial cells (17). Another study in CAD patients demonstrated down-regulation of Chast, HULC, and DICER1-AS1 in the peripheral blood samples (18). Expression of CASC11 has also been decreased in patients with CAD parallel with overexpression of TGF-β1. Functional studies revealed the impact of this lncRNA in the suppression of TGF-β1 expression in endothelial cells (19).

#### LncRNAs Alter Function of Endothelial Cells and Vascular Smooth Muscle Cells

**Figure 1:**
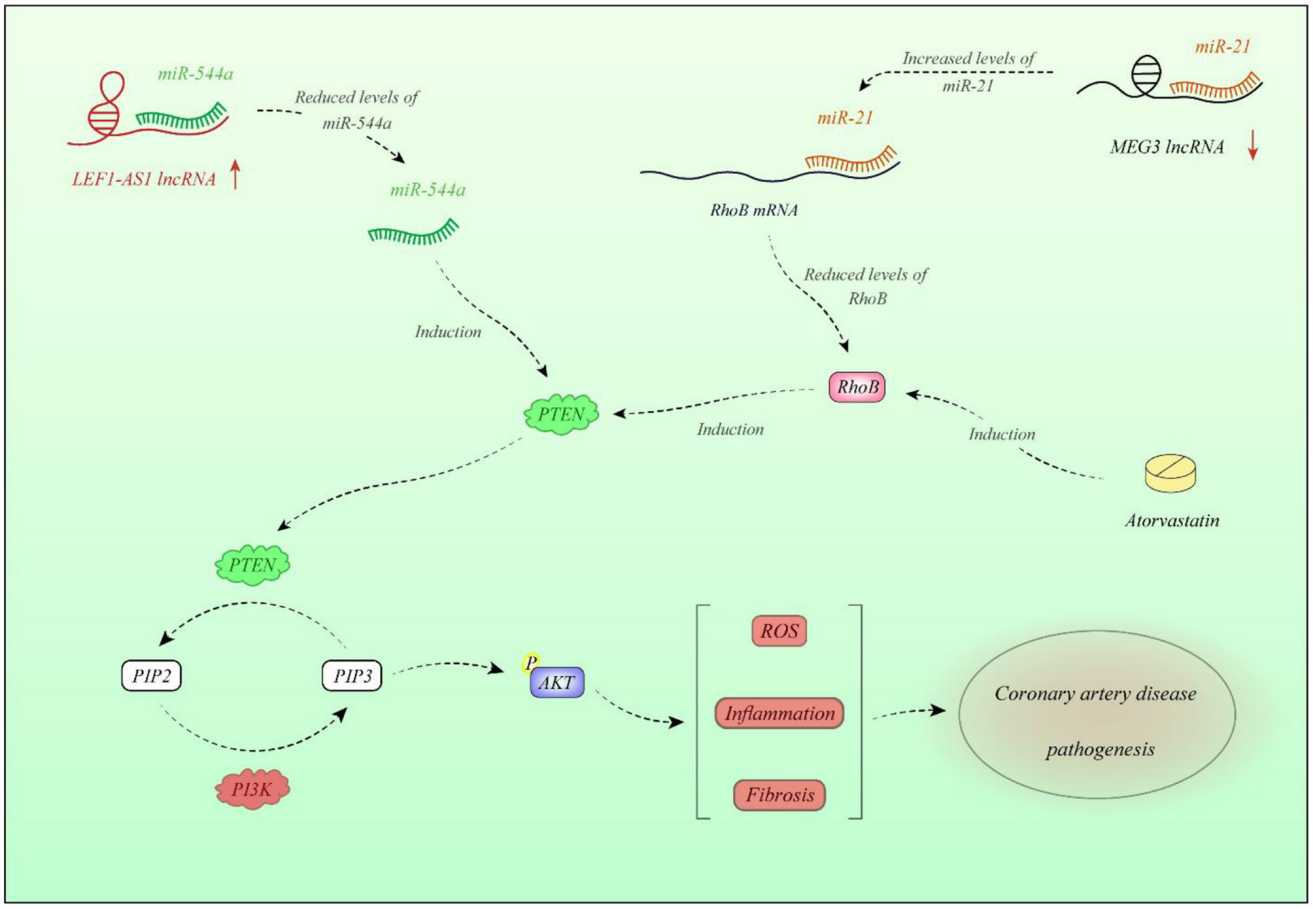
Expression of LEF1-AS1 is increased in plasma and tissues of CAD patients. LEF1-AS1 inhibits miR-544a, thus decreasing PTEN. This is because miR-544a increases PTEN levels. PTEN diminishes AKT activity. LEF1-AS1 contributes in enhancement of reactive oxygen species (ROS) formation, elevation of inflammatory responses, and fibrosis through miR-544a/PTEN axis (28). On the other hand, expression of MEG3 is decreased in CAD tissues compared with normal tissues. Down-regulation of MEG3 is associated with up-regulation of miR-21. miR-21 binds with 3′-UTR of RhoB and decreases its expression. RhoB has a role in activation of PTEN; therefore, MEG3 down-regulation is associated with the lower activity of PTEN (29). Atorvastatin has a regulatory role on PTEN through modulation of RhoB (30).

ANRIL is another up-regulated lncRNA in CAD patients as well as animal model of this disorder. Overexpression of ANRIL decreases expression of miR-181b and is associated with risk of CAD in the subpopulations of elderly patients with history of smoking, hypertension, and hyperlipidemia. Overexpression of ANRIL in human coronary endothelial cells has down-regulated miR-181b, increased p50/p65 expressions, enhanced viability of human coronary endothelial cells, and promoted release of inflammatory molecules and vascular-protecting proteins (20). Another study has revealed overexpression of ANRIL in patients with acute coronary syndrome in association with levels of monocyte chemoattractant protein-1 and IL-10. Notably, these proinflammatory cytokines are produced in reaction to dysfunction of endothelial cells. ANRIL silencing has enhanced cell proliferation and tubule development and suppressed induction of inflammatory responses and apoptosis of endothelial cells. Such effects were linked to ANRIL-mediated suppression of let-7b and its impacts on the TGF-βR1/Smad signaling (21). Another study has demonstrated higher levels of ANRIL and MIAT in the atherosclerotic arteries when compared to the non-atherosclerotic ones (22). ANRIL can also regulate growth of vascular smooth muscle cells via modulation of CDKN2A/B locus, which has a direct effect on the pathobiology of atherosclerosis (23). H19 in addition to its role in the regulation of immune responses can influence vascular smooth muscle cells. Expression of this lncRNA has been increased in the injured neointima and in human atherosclerotic plaques but is scarcely deceted in normal vessels. H19 sequesters let-7 family microRNAs (miRNAs), which are known to shield vascular smooth muscle cells from oxidative damage (12, 24). Expression of lnc-DC has been higher in patients with type 2 diabetes and CAD compared with diabetic patients without CAD. Such up-regulation has been accompanied by overexpression of STAT3. Yet, expression of these genes was not associated with the severity of CAD. Based on the observed correlations between expression of genes, authors have suggested the importance of JAK/STAT-related-lncRNAs in the pathogenesis of CAD (25). Expression of FAL1 has been shown to be elevated in CAD tissues and TNF-α-stimulated endothelial cells compared with normal and unstimulated cells. Up-regulation of FAL1 in endothelial cells has enhanced cell cycle progression, proliferation, and migration via modulation of PTEN/AKT pathway (26). In addition, HIF1a-AS1 partakes in the pathology of atherosclerosis via modulating apoptosis of vascular smooth muscle cells and endothelial cells (27). Expression of CASC11 has also been decreased in patients with CAD parallel with overexpression of TGF-β1. Functional studies revealed the impact of this lncRNA in the suppression of TGF-β1 expression in endothelial cells (19). Expression of LEF1-AS1 has been elevated in plasma and tissue samples of CAD patients, whereas expression of its target miRNA, i.e., miR-544a, has been decreased. LEF1-AS1 modulates proliferation and migration of smooth muscle cells via the miR-544a/PTEN route (28). Figure 1 shows the molecular cascade of contribution of LEF1-AS1 and MEG3 in CAD.

#### LncRNAs Regulate Lipid Metabolism

**Figure 2:**
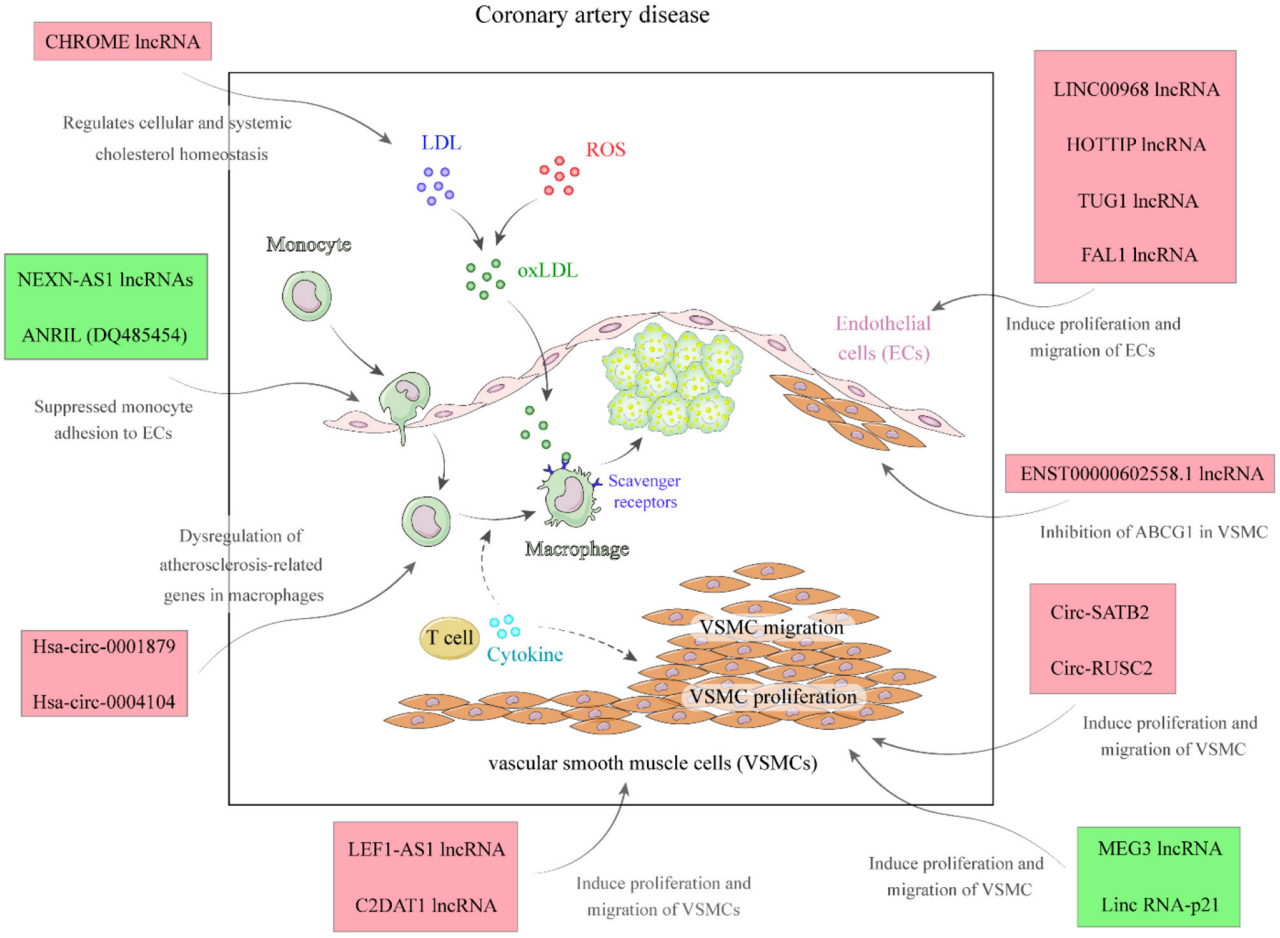
A summary of the role of different lncRNAs in the pathophysiology of CAD.

LincRNA-DYNLRB2-2 is an lncRNA whose expression is stimulated by Ox-LDL. This transcript enhances ABCA1-associated cholesterol efflux and suppresses inflammatory responses via GPR119 in macrophage originated foam cells (31). CHROME is another up-regulated lncRNA in CAD patients whose expression is altered by nutritional and cellular cholesterol levels via the sterol-activated liver X receptor transcription factors. This lncRNA enhances cholesterol secretion and HDL synthesis through suppression of the activity of a number of miRNAs. CHROME silencing in human hepatocytes and macrophages enhances expressions of miR-27b, miR-33a, miR-33b, and miR-128, thus decreasing the levels of their shared target genes, particularly ABCA1, which controls *de novo* synthesis of HDL (Figure 2) (32). Expression of FAL1 has been shown to be elevated in CAD tissues and TNF-α-stimulated endothelial cells compared with normal and unstimulated cells. Up-regulation of FAL1 in endothelial cells has enhanced cell cycle progression, proliferation, and migration via modulation of PTEN/AKT pathway (26). GAS5 is another down-regulated lncRNA in CAD. Enforced up-regulation of GAS5 in animal models of CAD has improved hyperlipidemia, reduced myocardial damage, suppressed apoptosis of cardiac cells, and diminished oxidative stress, inflammatory damage, and aberrant induction of the Wnt/β-catenin pathway in cardiac tissue (33). The function of up-regulated and down-regulated lncRNAs in CAD is summarized in Tables 1, 2, respectively.

**Table 1:** List of up-regulated lncRNAs in CAD (HCAECs, human coronary endothelial cells; HUVECs, human umbilical vein endothelial cells; VSMCs, vascular smooth muscle cells).

**LncRNA**	**Samples**	**Assessed cell lines**	**Interactions**	**Signaling pathway**	**Association with clinical properties**	**Function**	**References**
*ANRIL*	327 patients with CAD, SD rats	HCAECs HUVECs	miR-181b, NF-κB	NF-κB signaling pathway	Age >60 years, smoking history, hyperlipidemia, hypertension, cholesterol level, triglyceride (TG) level	Regulates viability and survival of HCAECs and modulates secretion of inflammatory agents from HCAECs through targeting miR-181b and regulating expression of NF-κB	(20)
*ANRIL*	111 CAD patients and 20 healthy controls	HUVECs	let-7b, TGF-βR1	TGF-βR1/Smad signaling pathway	–	Regulates HUVEC function by targeting let-7b and regulation of TGF-βR1/Smad signaling pathway activation	(21)
*ANRIL, MIAT*	Atherosclerotic coronary tissue specimens from 20 patients	–	–	–	–	Are up-regulated and may participate in pathogenesis of CAD	(22)
*lnc-DC*	37 patients with CAD and 36 patients without CAD	–	SOCS1, STAT3	JAK/STAT pathway	–	My be implicated in pathogenesis of CAD	(25)
*IFNG-AS1*	Plasma samples from 102 patients with CAD and 89 control subjects	–	–	–	Gensini score, hs-CRP, TNF-α, IL-6, and IL-10 levels	Its expression was associated with augmented risk of CAD, enhanced severity of disease, and increased inflammation	(15)
*LEF1-AS1*	Tissue specimens from 70 patients with coronary artery atherosclerosis and 30 healthy controls	VSMCs	miR-544a, PTEN	PTEN pathway	Patients survival	Promotes proliferation, invasion, and migration of smooth muscle cells through regulating miR-544a/PTEN axis	(28)
*H19*	Serum samples from 30 CAD patients and 30 healthy subjects	HCAEC	TGF-β1	–	–	Contributes to CAD pathogenesis through increasing expression TGF-β1	(13)
*FAL1*	15 CAD tissues and 15 normal arterial tissues	HUVECs	–	PTEN/AKT pathway	–	Enhances proliferation, migration, and cell cycle progression through activating PTEN/AKT pathway	(26)
*OTTHUMT00000387022*	246 CAD patients and 206 control subjects	THP-1	–	–	–	Its knockdown in THP-1 cells reduces release of proinflammatory cytokines from these cells	(34)
*LncPPARδ*	246 patients with CAD and 206 healthy subjects	THP-1	PPARδ, ADRP, ANGPTL4	–	–	Its knockdown influenced expression of PPARδ, ADRP and ANGPTL4	(14)
*C2dat1*	20 CAD tissues and 20	VSMC	miR-34a, SIRT1	–	–	Its overexpression enhances proliferation and migration of VSMC by regulating miR-34a/SIRT1 axis	(35)
*CHROME (ENSG00000223960)*	Plasma samples from 14 CAD patients and 33 healthy volunteers as controls, 127 atherosclerotic plaques from CAD patients and 10 normal arteries, male African green monkeys	THP-1, HEK293T, HepG2	miR-33b, miR-27b, miR-128, miR-33a, ABCA1	–	–	Its knockdown inhibits HDL biogenesis and cholesterol efflux through modulating expression of miR-33b, miR-27b, miR-128, miR-33a, and ABCA1	(32)
*THRIL*	Plasma samples from 220 patients with coronary heart disease and 200 control individuals	–	–	–	Gensini score, diabetes mellitus, fasting blood glucose, CRP, TNF-α, MACE accumulating rate	Can be a potential risk factor for prediction of coronary heart disease	(36)
*HOTTIP*	15 CAD tissues and 15 normal arterial tissues	HU-VECs	β-catenin, c-Myc	Wnt/β-catenin pathway	–	Its up-regulation enhances proliferation and migration of endothelial cells by regulating β-catenin expression and activation of Wnt/β-catenin pathway	(37)
*ENST00000602558.1*	–	VSMCs	ABCG1	–	–	Its overexpression decreases expression of ABCG1 and thus lower cholesterol efflux and HDL biogenesis	(38)
*LINC00968*	20 CAD tissues and 20 normal arterial tissues	Endothelial cell	miR-9-3p	–	–	Its overexpression promotes migration and proliferation of endothelial cells through targeting miR-9-3p	(39)
*TUG1*	15 CAD tissues and normal arterial tissues	HUVECs	β-catenin, c-Myc	Wnt pathway	–	Enhances migration, proliferation, and cell cycle progression in HUVECs	(40)

**Table 2:** List of down-regulated lncRNAs in CAD (HCAEC, human coronary endothelial cells; HUVEC, human umbilical vein endothelial cells).

**LncRNA**	**Samples**	**Cell lines**	**Interactions**	**Signaling pathway**	**Association with clinical properties**	**Function**	**References**
*GAS5*	Plasma samples from 30 CAD patients and 30 healthy controls	HCAECs	p-mTOR	mTOR pathway	–	Its down-regulation increases phosphorylated mTOR levels, but its up-regulation has reverse effects	(41)
*GAS5*	Serum samples from 102 CAD patients and 98 control subjects, 72 y Sprague–Dawley rats established as CAD model	–	–	Wnt/β-catenin signaling pathway	CK-MB, Troponin I, Gensini score	Inhibits apoptosis of cardiomyocyte, oxidative stress, and inflammatory damage in CAD rat models; reduces myocardial damages in these rats	(33)
*NEXN-AS1*	Atherosclerotic arterial samples from patients and normal individuals, blood samples from 113 CAD patients, 69 myocardial infarction, 40 heart failure, and 40 healthy subjects, ApoE^−/−^ mice, NEXN^+/−^ mice	THP-1, HUVECs, VSMCs	NEXN, BAZ1A	–	–	Interacts with BAZ1A and contributes to elevation of NEXN expression	(17)
*ANRIL (DQ485454)*		HCAECs, HUVECs	CLIP1, EZR, LYVE1	–	–	Its overexpression suppresses TEM process in monocyte migration of endothelial cells and adhesion of monocytes to endothelial cells	(16)
*Chast*	Blood samples from 50 premature CAD patients and 50 age- and gender-matched healthy volunteers as controls	–	–	–	FBS levels	May be implicated in CAD development	(18)
*HULC*	Blood samples from 50 premature CAD patients and 50 age- and gender-matched healthy volunteers as controls	–	–	–	Age, FBS, TG	Has diagnostic value for distinguishing CAD patients from healthy individuals	(18)
*DICER1-AS1*	Blood samples from 50 premature CAD patients and 50 age- and gender-matched healthy volunteers as controls	–	–	–	FBS levels, TG, TG/HDL ratio	Has diagnostic value for distinguishing CAD patients from healthy individuals	(18)
*MEG3*	40 CAD tissues and 35 control tissues	VSMC	miR-26a, Smad1	–	–	Its overexpression represses proliferation and induces apoptosis in VSMCs by targeting miR-26a and increasing expression of Smad1	(42)
*MEG3*	15 CAD tissues and 15 normal arterial tissues	HUVECs	miR-21, RhoB, PTEN	–	–	Its overexpression suppressed proliferation and migration through targeting miR-21 and regulation of RhoB and PTEN expression	(29)
*lincRNA-p21*	Blood samples from 12 CAD patients and 8 control subjects, ApoE^−/−^ mice	HA-VSMC, RAW264.7	MDM2, p53	–	–	Regulates proliferation and apoptosis in vascular smooth muscle cell by interacting with MDM2 and modulation of p53 activity	(43)
*CASC11*	Plasma samples from 82 CAD patients and 82 age- and gender-matched healthy individuals	HCAECs	TGF-β1	–	Patients survival	Its overexpression reduces expression of TGF-β1 in HCAECs	(19)

#### Prognostic Value of LncRNAs in CAD

LncRNAs can be used for evaluation of prognosis of CAD patients. For instance, a long-term follow-up study has demonstrated correlation between down-regulation of CASC11 and poor survival of patients with CAD (19). On the other hand, overexpression of LEF1-AS1 and ANRIL has been shown to be correlated with poor clinical outcome of patients with CAD (28, 44). Kaplan–Meier analysis has also demonstrated association between ANRIL and LEF1-AS1 overexpression and short overall survival in CAD patients (28, 44). Table 3 reviews the studies that appraised the prognostic role of lncRNAs in CAD.

**Table 3:** Prognostic role of lncRNAs in CAD (OS, overall survival).

**LncRNA**	**Samples**	**Kaplan–Meier analysis**	**Univariate analysis**	**Multivariate analysis**	**References**
*CASC11*	Plasma samples from 82 CAD patients and 82 age- and gender-matched healthy individuals	Its low expression was associated with decreased overall survival in CAD patients	–	–	(19)
*ANRIL*	327 patients with CAD	–	Its expression was correlated with CAD patients survival	Its expression can be an independent predictor of CAD patients' survival	(20)
*LEF1-AS1*	Tissue specimens from 70 patients with coronary artery atherosclerosis and 30 healthy controls	High expression of *LEF1-AS1* was associated with poor OS.	–	–	(28)
*ANRIL*	Plasma samples from 125 CAD patients and 105 control individuals	High expression of *ANRIL* was associated with shorter OS in CAD patients.	–	–	(44)

#### Diagnostic Value of LncRNAs in CAD

Blood or serum levels of some lncRNAs can be used as diagnostic markers in CAD. The best diagnostic value has been reported for H19 where the receiver operating characteristic (ROC) curves showed the diagnostic power of 0.9367, signifying H19 as a suitable marker for CAD (13). Cai et al. (14) have profiled lncRNAs in circulating peripheral blood monocytes and plasma samples of CAD patients and healthy subjects. Their preliminary results demonstrated possible biomarker role for CoroMarker, BAT5, and IL21R-AS1 lncRNAs. The verification step in the larger cohort of CAD patients supported the biomarker role of CoroMarker. This lncRNA could differentiate CAD patients from healthy subjects with accuracy of 0.920 and in an independent manner from identified CAD risk factors and other cardiovascular disorders (14). Another study demonstrated the accuracy of 0.90 and 0.87 for HULC and DICER1-AS1, respectively, in differentiation between CAD patients and healthy individuals (18). In addition, up-regulation of IFNG-AS1 in CAD patients could be used to forecast the risk of CAD with accuracy of 0.755 (15). Table 4 summarizes the points regarding the diagnostic significance of lncRNAs in CAD.

**Table 4:** Diagnostic role of lncRNAs in CAD.

**LncRNA**	**Expression pattern**	**Sample**	**Type of marker**	**ROC curve analysis**			**References**
				**Sensitivity**	**Specificity**	**Area under the curve (AUC)**	
*GAS5*	Down-regulated	Serum samples from 102 CAD patients and 98 control subjects	Diagnostic marker	86.7%	86.5%	0.889	(33)
*HULC*	Down-regulated	Blood samples from 50 premature CAD patients and 50 age- and gender-matched healthy volunteers as controls	Diagnostic marker	–	–	0.90	(18)
*DICER1-AS1*	Down-regulated	Blood samples from 50 premature CAD patients and 50 age- and gender-matched healthy volunteers as controls	Diagnostic marker	–	–	0.87	(18)
*CASC11*	Down-regulated	Plasma samples from 82 CAD patients and 82 age- and gender-matched healthy individuals	Diagnostic marker	–	–	0.90	(19)
*ANRIL (EU741058)*	Down-regulated	Blood samples from 50 CAD patients and 50 healthy volunteers	Diagnostic marker	82%	69%	–	(45)
*IFNG-AS1*	Up-regulated	Plasma samples from 102 patients with CAD and 89 control subjects	Diagnostic marker (for prediction of CAD risk)	–	–	0.755	(15)
*H19*	Up-regulated	Serum samples from 30 CAD patients and 30 healthy subjects	Diagnostic marker	–	–	0.9367	(13)
*OTTHUMT00000387022*	Up-regulated	246 CAD patients and 206 control subjects	Diagnostic marker	–	–	0.920	(34)
*LncPPARδ*	Up-regulated	246 patients with CAD and 206 healthy subjects	Diagnostic marker	–	–	0.727	(14)
*LncPPARδ* along with CAD risk factors	Up-regulated	246 patients with CAD and 206 healthy subjects	Diagnostic marker	–	–	0.785	
*THRIL*	Up-regulated	Plasma samples from 220 patients with coronary heart disease and 200 control individuals	Diagnostic marker	–	–	0.869	(36)
*ANRIL*	Up-regulated	Plasma samples from 125 CAD patients and 105 control individuals	Diagnostic marker	–	–	0.789	(44)
*AC100865.1*	Up-regulated	Plasma samples from 256 patients with CAD and 222 healthy individuals	Diagnostic marker	–	–	0.795	(46)
*ENST00000444488.1*	–	Blood samples [peripheral blood mononuclear cells (PBMCs)] from 505 CAD patients and 343 male individuals as controls	Diagnostic marker [distinguishing patients with acute myocardial infarction (AMI) form non-AMI patients]	–	–	0.758	(47)
*ENST00000444488.1*	–	Blood samples (PBMCs) from 505 CAD patients and 343 male individuals as controls	Diagnostic marker (distinguishing patients with CAD from controls)	–	–	0.799	
*uc010yfd.1*	–	Blood samples (PBMCs) from 505 CAD patients and 343 male individuals as controls	Diagnostic marker (distinguishing patients with CAD from controls)	–	–	0.779	
*ENST00000444488.1 uc010yfd.1*	–	Blood samples (PBMCs) from 505 CAD patients and 343 male individuals as controls	Diagnostic marker (distinguishing patients with CAD from controls)	–	–	0.851	
*ENST00000444488.1 uc010yfd.1* along with age, BMI, glucose, and HDL	–	Blood samples (PBMCs) from 505 CAD patients and 343 male individuals as controls	Diagnostic marker (distinguishing patients with CAD from controls)	–	–	0.902	
*H19*	Up-regulated	Plasma samples from 300 CAD patients and 180 control individuals	Diagnostic marker	–	–	0.631	(48)
*LIPCAR*	Up-regulated	Plasma samples from 300 CAD patients and 180 control individuals	Diagnostic marker	–	–	0.722	
*KCNQ1OT1*	Up-regulated	Blood samples (PBMCs) from 20 patients with CAD and 20 individuals without CAD	Diagnostic marker	–	–	0.865	(49)
*HIF1A-AS2*	Up-regulated	Blood samples (PBMCs) from 20 patients with CAD and 20 individuals without CAD	Diagnostic marker	–	–	0.852	
*APOA1-AS*	Up-regulated	Blood samples (PBMCs) from 20 patients with CAD and 20 individuals without CAD	Diagnostic marker	–	–	0.967	
*KCNQ1OT1* *HIF1A-AS2* *APOA1-AS*	Up-regulated Up-regulated Up-regulated	Blood samples (PBMCs) from 20 patients with CAD and 20 individuals without CAD	Diagnostic marker	–	–	0.990	
*ENST00000512246.1*	Up-regulated	Blood samples from 173 CAD patients and 151 healthy controls	Diagnostic marker	0.833	0.7	0.804	(50)
*TCONS_00023843*	Up-regulated	Blood samples from 173 CAD patients and 151 healthy controls	Diagnostic marker	0.767	0.567	0.69	
*NR_028044.1*	Up-regulated	Blood samples from 173 CAD patients and 151 healthy controls	Diagnostic marker	0.6	0.833	0.739	
*TCONS_00029157*	Up-regulated	Blood samples from 173 CAD patients and 151 healthy controls	Diagnostic marker	0.667	0.833	0.769	
*MIAT*	Up-regulated	Blood samples from 110 CAD patients and 117 volunteers as controls	Diagnostic marker	95.5%	72.7%	0.888	(51)

#### LncRNAs Polymorphisms and CAD

A number of functional single-nucleotide polymorphisms (SNPs) in lncRNAs have been associated with susceptibility to CAD. ANRIL has been the mostly assessed lncRNA in this regard. For instance, rs1330049, rs2383206, rs10757278, and rs10757274 SNPs have been associated with risk of CAD in Asians (52). On the other hand, rs2383207 and rs1333049 SNPs of ANRIL have not been associated with CAD risk in Han Chinese (53). In addition, rs1333040 and rs1004638 SNPs of ANRIL have not been associated with this disorder in Iranian population (54). H19 is another lncRNA whose association with risk of CAD has been assessed in some populations. Hu et al. (55) have reported an association between H19 rs2735971 and rs3024270 SNPs and susceptibility to CAD in a Chinese population, suggesting the significance of these SNPs as markers for prediction of risk of CAD in this population. Other SNPs within LINC00841, MALAT1, and lincRNA-p21 have been associated with risk of CAD in some ethnic groups (Table 5).

**Table 5:** LncRNAs polymorphisms and CAD.

**LncRNA**	**Polymorphism**	**Samples**	**Population**	**Assay method**	**Association**	**References**
*ANRIL*	rs1330049, rs2383206, rs10757278, rs10757274	Blood samples from 1,034 CAD patients and 1,034 healthy subjects	Asian Indians	TaqMan allelic discrimination assay	All of these SNPs were associated with CAD risk	(52)
*ANRIL*	rs2383207, rs1333049	Blood samples from 550 CAD patients, 550 patients with ischemic stroke, and 550 healthy individuals	Han Chinese	Sequenom MassARRAY on an Agena platform	There was no association between theses SNPs and CAD predisposition	(56)
*ANRIL*	rs496892, rs7865618	Blood samples from 100 patients with periodontitis (PD) and CAD and 100 healthy volunteers as controls	South Indian population	ARMS-PCR, PCR-RFLP	Both of these polymorphisms were associated with elevated risk of PD and CAD Also rs496892-rs7865618 A-G and rs496892-rs7865618 G-G haplotypes were associated with increased risk of PD and CAD	(53)
*ANRIL*	rs564398, rs4977574, rs2891168, rs1333042	Blood samples from 250 patients with CAD and 252 age-matched control subjects	Saudi Population	TaqMan assay	Distribution of these SNPs were different in CAD group and control group	(57)
*ANRIL*	rs1333040, rs1004638	Blood samples from 200 patients with CAD 110 healthy subjects	Iranian	PCR-RFLP	There was no association between these SNPs and CAD susceptibility	(54)
*H19*	rs2735971, rs3024270, rs2839698	Blood samples from 366 CAD patients and 366 matched control individuals	Chinese	KASP platform	Genotypes of rs2735971 and rs3024270 were associated with reduced risk of CAD Also rs2735971-rs2839698-rs3024270 A-C-C haplotype was associated with decreased risk of CAD.	(55)
*H19*	rs217727, rs2067051	Blood samples from 701 patients with CAD and 873 age- and gender-matched control individuals	Chinese	TaqMan real time PCR	A allele of rs2067051 was associated with reduced risk of CAD, but T allele of rs217727 was correlated with elevated CAD risk.	(58)
*LINC00841*	rs1870634	Blood samples from 155 patients with CAD and 112 age- and sex-matched subjects without CAD	Iranian	real-time PCR-HRM	GG genotype of rs1870634 was correlated with augmented risk of CAD	(59)
*MALAT1*	rs619586	Blood samples from 508 patients with CAD and 562 age-, sex-, and ethnicity-matched control individuals	Chinese	TaqMan allelic discrimination assay	G allele and AG/GG genotypes of rs619586 were associated with decreased risk of CAD	(60)
*lincRNA-p21*	rs9380586, rs4713998, rs6930083, rs6931097	Blood samples from 615 CAD patients and 655 control subjects	Han Chinese	PCR-LDR	rs9380586-rs4713998-rs6930083-rs6931097 G-A-A-G haplotype was associated with decreased CAD risk	(61)
*SENCR*	rs555172	Blood samples from 150 CAD patients and 149 healthy controls	Iranian	ARMS-PCR	There was no association between rs555172 polymorphism and CAD predisposition	(62)

## CircRNAs and CAD

### Role of CircRNAs in CAD

A comprehensive circRNA profiling in CAD patients has shown up-regulation of 624 circRNAs and down-regulation of 171 circRNAs in these patients compared with healthy subjects. Subsequent validation in a larger cohort of patients supported up-regulation of hsa_circ_0001879 and hsa_circ_0004104 in CAD patients. Remarkably, up-regulation of hsa_circ_0004104 has led to aberrant expression of atherosclerosis-associated genes in macrophages (63). Another high-throughput study of circRNAs expression in CAD patients has shown up-regulation of 18 circRNAs, whereas down-regulation has been shown in six circRNAs. Subsequently, authors have reported the role of nine circRNAs in the enhancement of TRPM3 expression through suppression of hsa-miR-130a-3p (64). Expression of circ-SATB2 has been shown to be increased in proliferative vascular smooth muscle cells in association with down-regulation of miR-939. Circ-SATB2 was able to augment expression of a target of miR-939, namely, STIM1. Up-regulation of circ-SATB2 decreases expression of SM22-α, a marker of contractile vascular smooth muscle cells. Functional studies verified the role of this circRNA in the regulation of differentiation, proliferation, apoptosis, and migration of vascular smooth muscle cells through enhancing STIM1 expression (65). Expression of circZNF609 has been shown to be reduced in peripheral blood leukocytes of CAD patients in correlation with levels of CRP and lymphocyte counts. Forced overexpression of circZNF609 has enhanced production of inflammatory cytokines IL-6 and TNF-α, while enhancing expression of IL-10. These effects are possibly mediated through sponging miRNAs (66). In an eminent study, Guo et al. (67) have assessed levels of hsa_circ_0002984, hsa_circ_0010283, and hsa_circ_0029589 in human peripheral blood mononuclear cell–originated macrophages from CAD patients and evaluated the consequences of overexpression or silencing of these circRNAs. Authors have reported down-regulation of hsa_circ_0029589 in macrophages, whereas up-regulation of the N6-methyladenosine levels of hsa_circ_0029589 in macrophages of patients with acute coronary syndrome has been shown. Notably, up-regulation of IRF-1 has diminished the expression of hsa_circ_0029589, but surged its m6A levels. Therefore, IRF-1 has been shown to enhance macrophage pyroptosis and inflammatory responses in acute coronary syndrome and atherosclerotic patients by obstructing circ_0029589 via increasing its m6A modifications (67). Tables 6, 7 show the list and function of up-regulated and down-regulated circRNAs in CAD, respectively.

**Table 6:** List of up-regulated circRNAs in CAD.

**CircRNAs**	**Samples**	**Assessed cell line**	**Gene/protein interaction**	**Association with clinical features**	**Function**	**References**
*Hsa_circ_0004104*	Blood samples from 436 male patients with CAD 297 male control subjects	THP-1	–	HDL-C levels	Its up-regulation was implicated in CAD pathogenesis and dysregulates proatherosclerotic and antiatherosclerotic genes expression	(63)
*circ-SATB2*	–	VSMCs	STIM1	–	Regulates proliferation, migration, and apoptosis in VSMCs by up-regulating expression of STIM1	(65)
*hsa_circ_0089378* *hsa_circ_0083357* *hsa_circ_0082824* *hsa_circ_0068942* *hsa_circ_0057576* *hsa_circ_0054537* *hsa_circ_0051172* *hsa_circ_0032970* *hsa_circ_0006323*	651 CAD patients and 287 control subjects	–	hsa-miR-130a-3p	–	Theses circRNAs suppress expression of hsa-miR-130a-3p and thus up-regulate expression of TRPM3 in CAD patients	(64)
*circ_RUSC2*	–	VSMCs	SYK	–	Promotes migration and invasion of VSMCs by up-regulating SYK expression	(68)

**Table 7:** List of down-regulated circRNAs in CAD.

**CircRNAs**	**Samples**	**Assessed cell line**	**Association with clinical features**	**Function**	**References**
*circZNF609*	Blood samples from (peripheral blood leukocytes) 330 patients with CAD and 209 control individuals	RAW264.7	C-reactive protein levels, lymphocyte counts	Its overexpression contributes to decreased expression of IL-6 and TNF-α and increased expression of IL-10	(66)
*hsa_circ_0029589*	Blood samples (PBMCs) from 24 patients with clinical presentation of chest pain, 16 patients with stable angina, and 28 patients with acute coronary syndrome	–	–	Its overexpression decreased pyroptosis of macrophages	(67)

### Diagnostic Value of CircRNAs in CAD

ROC curve analysis has shown that hsa_circ_0001879 and hsa_circ_0004104 could differentiate CAD patients from healthy subjects with diagnostic values of 0.703 and 0.700, respectively. Notably, combination of expression levels of these circRNAs, in conjunction with CAD risk factors, could enhance the diagnostic power (63). Expression of circZNF609 has been shown to be reduced in peripheral blood leukocytes of CAD patients in correlation with levels of CRP and lymphocyte counts. In addition, down-regulation of circZNF609 has been associated with higher risks of CAD with accuracy of 0.761 (66). In a high-throughput circRNA profiling, Zhang et al. have demonstrated differential expression of 22 circRNAs between CAD patients and healthy subjects. Among these circRNAs, hsa_circ_0124644 has been shown to have the highest AUC value. Validation of their results in a larger cohort of CAD patients showed the diagnostic power of 0.769 for this circRNA (69). Combination of expression profile of certain circRNAs with conventional CAD risk factors has enhanced the diagnostic power (Table 8).

**Table 8:** Diagnostic Role of circRNAs in CAD.

**CircRNAs**	**Expression pattern**	**Samples**	**ROC curve analysis**			**References**
			**Sensitivity**	**Specificity**	**AUC**	
*Hsa_circ_0004104*	Up-regulated	Blood samples from (PBMCs) 436 male patients with CAD 297 male control subjects	–	–	0.700	(70)
*Hsa_circ_0001879*	Up-regulated	Blood samples from (PBMCs) 436 male patients with CAD 297 male control subjects	–	–	0.703	
*Hsa_circ_0004104* *Hsa_circ_0001879*	Up-regulated Up-regulated	Blood samples from (PBMCs) 436 male patients with CAD 297 male control subjects	–	–	0.742	
*Hsa_circ_0004104* *Hsa_circ_0001879* together with smoking, TC and serum creatinine	Up-regulated Up-regulated	Blood samples from (PBMCs) 436 male patients with CAD 297 male control subjects	–	–	0.832	
*Hsa_circ_0124644*	Up-regulated	Blood samples from 179 CAD patients and 157 control individuals	0.861	0.626	0.769	(69)
*Hsa_circ_0124644* along with smoking, hypertension, DM, LDL, and TC	Up-regulated	Blood samples from 179 CAD patients and 157 control individuals	0.759	0.704	0.804	
*Hsa_circ_0124644* *Hsa_circ_0098964*	Up-regulated Up-regulated	Blood samples from 179 CAD patients and 157 control individuals	0.825	0.730	0.811	
*Hsa_circ_0124644* *Hsa_circ_0098964* along with smoking, hypertension, DM, LDL, and TC	Up-regulated Up-regulated	Blood samples from 179 CAD patients and 157 control individuals	0.832	0.696	0.843	
*hsa_circ_0005540*	Up-regulated	Plasma exosomes 108 patients with CAD and 89 subjects without CAD	–	–	0.853	(71)
*circ-YOD1*	Up-regulated	Analysis of 7 CAD-related microarray datasets	–	–	0.824	(72)
*circZNF609*	Down-regulated	Blood samples from (peripheral blood leukocytes) 330 patients with CAD and 209 control individuals	–	–	0.761	(66)

### CircRNA Polymorphisms and CAD

Finally, SNPs within circRNAs can alter the risk of CAD. Zhou et al. (73) have appraised the impact of two SNPs at the circFOXO3 flanking introns in the development of CAD in a Chinese population. They reported the association between the rs12196996 G allele and elevated susceptibility to CAD. Moreover, such association was more remarkable among younger individuals and non-smokers. The haplotype rs12196996G-rs9398171C has also been associated with susceptibility to CAD. Functionally, the rs12196996 GG genotype conferred lower amounts of circFOXO3 expression (73).

## Discussion

CAD is a pathogenic condition in which several cell types and molecules are involved. In fact, this condition results from abnormal interactions between numerous cell types in the artery walls. The main participating factors in this process are accumulation of lipid deposits, endothelial cell dysfunction, macrophage induction, and changes in smooth muscle cells (5). Non-coding RNAs can influence almost every aspect of this pathogenic process. Dysregulation of several lncRNAs and circRNAs has been noted in CAD. ANRIL has been among the most assessed lncRNAs in CAD. Whereas, most studies have demonstrated up-regulation of ANRIL in patients with CAD or animal models of CAD (20, 22), a single study has reported down-regulation of certain transcript of ANRIL in CAD coronary arteries as compared with samples obtained from non-CAD arteries (16). Therefore, transcript variants of lncRNAs might have different tissue specificity and diverse functional roles. Such detailed analysis of transcript variants has not been performed for other lncRNAs in the context of CAD.

Dysregulated lncRNAs in CAD patients have functional interactions with Wnt/β-catenin, NF-κB, TGF-βR1/Smad, JAK/STAT, PTEN/AKT, and mTOR signaling pathways. Therefore, these signaling pathways are putative targets for therapeutic manipulations in CAD. Comprehensive studies are needed to explore the interactions between all mentioned lncRNAs and these pathways to find the most appropriate lncRNA for therapeutic interventions. The lncRNAs with the most robust interactions with higher numbers of these pathways are probably the most suitable targets. Moreover, CAD-related lncRNAs have interactions with a number of miRNAs such as miR-181b, let-7b, miR-544a, miR-34a, miR-33b, miR-26a, miR-27b, miR-21, miR-128, and miR-33a, suggesting the complicated interactions between diverse non-coding RNAs in the context of CAD. Identification of this multifaceted interaction network is a prerequisite for the development of anti-CAD strategies. Such network has a practical significance in the design of prognostic or diagnostic panels.

Functional studies have confirmed the causal effects of lncRNAs/circRNAs in the pathogenesis of CAD, as the ectopic expression of these transcripts in human endothelial cells has led to dysregulation of proliferation, cell cycle transition, and migration in the favor of CAD development. Moreover, altered expression of these transcripts in immune cells has provoked immune responses and suppressed anti-inflammatory cytokines. A number of additional lncRNAs/circRNAs have functional impact on deposition of lipid in the vessels as demonstrated by *in vivo* experiments. The synergic effects of these transcripts in the pathobiology of CAD should also be evaluated in functional studies through establishment of double-knockout models.

Some SNPs within lncRNAs have been related with risk of CAD in certain populations. However, the results of these studies have not been replicated in other populations. Therefore, these data are not sufficient to propose these SNPs as markers for CAD in a pan-ethnic scale. Moreover, assessment of haplotypes of these SNPs and their association with risk of CAD would provide more reliable results in this regard.

In addition, circRNAs could partake in the development of CAD via modulation of proliferation, differentiation, or apoptosis in CAD-related cells such as vascular smooth muscle cells. Such roles may be mediated through modulation of expression of several target genes, particularly miRNAs. Similar to lncRNAs, circRNAs can serve as molecular sponges for miRNAs. Expression levels of some circRNAs can be used as diagnostic markers in CAD.

In brief, CAD has been associated with dysregulation of numerous lncRNAs and circRNAs. Moreover, these kinds of non-coding transcripts can be used as markers for prediction of risk of CAD and disease course. The potential of these transcripts as therapeutic targets should be appraised in upcoming investigations. The most important limitation of studies that assessed the functional role of lncRNAs/circRNAs in the pathogenesis of CAD is that they are mostly dependent on cell line studies or animal studies whose generalization to human subjects is not easy. Moreover, the consequences of the observed altered expression of lncRNAs/circRNAs in human subjects should be assessed in follow-up studies. Finally, the association between lncRNAs/circRNAs signature and response to the therapeutic option in CAD patients including coronary artery bypass graft, percutaneous coronary intervention, and medical therapies should be assessed in upcoming studies.

## Author Contributions

MT and SG-F wrote the draft and revised it. MG collected the data and designed the tables. MT designed the figures. All the authors approved the submitted version.

## Conflict of Interest

The authors declare that the research was conducted in the absence of any commercial or financial relationships that could be construed as a potential conflict of interest.
